# Dissociations between the horizontal and dorsoventral axes in body-size perception

**DOI:** 10.1111/ejn.12187

**Published:** 2013-03-20

**Authors:** Teruo Hashimoto, Atsushi Iriki

**Affiliations:** 1 Laboratory for Symbolic Cognitive Development, RIKEN BSIWako City, Japan; 2 Centre for Advanced Research on Logic and Sensibility, Keio UniversityTokyo, Japan

**Keywords:** body thickness, functional MRI, fusiform body area, SII, somatosensory cortex

## Abstract

Body size can vary throughout a person's lifetime, inducing plasticity of the internal body representation. Changes in horizontal width accompany those in dorsal-to-ventral thickness. To examine differences in the perception of different body axes, neural correlates of own-body-size perception in the horizontal and dorsoventral directions were compared using functional magnetic resonance imaging. Original and distorted (−30, −10, +10 and +30%) images of the neck-down region of their own body were presented to healthy female participants, who were then asked whether the images were of their own body or not based explicitly on body size. Participants perceived body images distorted by −10% as their own, whereas those distorted by +30% as belonging to others. Horizontal width images yielded slightly more subjective own-body perceptions than dorsoventral thickness images did. Subjective perception of own-body size was associated with bilateral inferior parietal activity. In contrast, other-body judgments showed pre-supplementary motor and superior parietal activity. Expansion in the dorsoventral direction was associated with the left fusiform gyrus and the right inferior parietal lobule, whereas horizontal expansions were associated with activity in the bilateral somatosensory area. These results suggest neural dissociations between the two body axes: dorsoventral images of thickness may require visual processing, whereas bodily sensations are involved in horizontal body-size perception. Somatosensory rather than visual processes can be critical for the assessment of frontal own-body appearance. Visual body thickness and somatosensory body width may be integrated to construct a whole-body representation.

## Introduction

The human body can be described using three axes: vertical (height); horizontal (width); and dorsoventral (thickness). Height is essentially stable throughout adulthood, but the other dimensions can vary. Although body width and thickness are in the same transverse direction in two dimensions for upright posture, body thickness differs from width in several ways: it is generally less than width (except for the feet), and viewing the body from the side is relatively infrequent when compared with viewing the body from the front. Accordingly, the dorsoventral view of thickness may require precise and fine visual processing. Visual perception of own-body width has been examined in normal subjects and in patients with eating disorders (Probst *et al*., 1998; Johnstone *et al*., 2008). However, the perception of dorsoventral thickness remains to be elucidated.

Sensorimotor representation of a person's own body parts facilitates own-body recognition (Frassinetti *et al*., 2011). Patients with sensory neuropathy have difficulty in controlling their limbs without exhibiting motor deficits (Sainburg *et al*., 1995). Perceptions of the size and shape of limbs are distorted by regional anesthesia, which interrupts somatosensory inputs (Paqueron *et al*., 2003; Turker *et al*., 2005). Patients with anorexia nervosa experience a disturbance in both visual and tactile own-body image (Keizer *et al*., 2011), even though their general size perceptions are normal (Cash & Deagle, 1997). Thus, tactile and proprioceptive inputs are necessary for body perception; however, no afferent signals are directly responsible for body size or shape perception (Longo *et al*., 2010).

Own-body perception is impaired by parietal lesions. Patients with anorexia nervosa and those with right parietal lesions overestimate their horizontal shoulder width (Nico *et al*., 2010). Somatoparaphrenia, a delusion of disownership of left-sided body parts, is associated with right parietal lesions (Vallar & Ronchi, 2009). Illusory own-body perceptions are related to pathological activity in the right temporoparietal junction (Blanke & Mohr, 2005) and parieto-insular vestibular cortex (PIVC; Lopez & Blanke, 2011). Therefore, the parietal cortex is critical for integrating visual and somatosensory inputs necessary for accurate body perception (Lewis & Van Essen, 2000; Avillac *et al*., 2005).

The perception of a horizontally distorted (fat or thin) body image, rather than the real body image, is associated with enhanced activity in the inferior parietal lobule (IPL) and fusiform gyrus (Miyake *et al*., 2010). The fusiform body area (FBA) is associated with body detection, whereas the primary somatosensory area (SI), intraparietal sulcus and IPL show greater activity when someone identifies the frontal view of their own body (Hodzic *et al*., 2009). These results suggest cognitive and conceptual involvement in own-body perception. Furthermore, the illusory sensation of waist-shrinking with tendon vibration is correlated with activity in the somatosensory cortex and intraparietal sulcus (Ehrsson *et al*., 2005). These results further suggest that own-body image can be an integrative representation.

In this study, we investigated neural correlates of the perception of body width and thickness by using distorted own-body images. The neural mechanism associated with discrimination between subjective own-body image perception and other-body image perception was also examined.

## Materials and methods

### Participants

Eleven healthy right-handed female subjects aged 19–27 years (mean ± standard deviation, 21.1 ± 2.3 years) participated in this study. The mean body mass index was 19.5 ± 1.3 kg/m^2^. Young female participants were exclusively recruited because they were expected to be highly body conscious (Wardle *et al*., 2006). Written informed consent was obtained from each participant, and the experiment was conducted in accordance with the Declaration of Helsinki and was approved by the Ethical Committee of Keio University, Japan.

### Materials

**FIG. 1:**
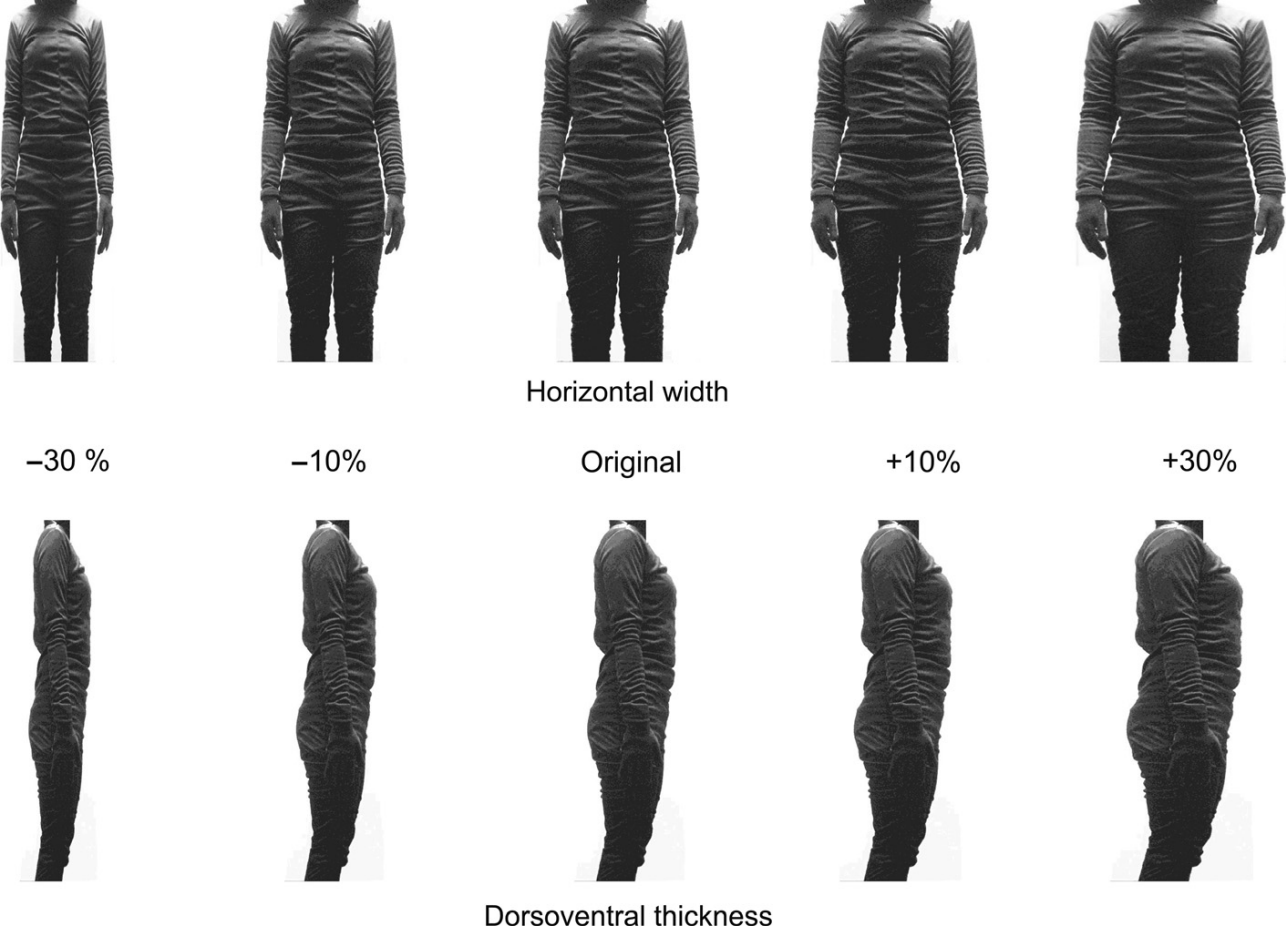
Sample of body images. Horizontal width (upper) and dorsoventral thickness (bottom) images were shown. Original images of each participant were used, and those images were distorted with vertical size fixed. Participants were informed that body images of their own and others would be presented, and they were asked to judge whether the images were of their own body or not based on the body size.

Color photographs of the participants' own bodies (neck to knees) were used (Fig. 1). Participants wore a black one-piece suit and stood in front of a white wall. Under the black suit, participants wore a T-shirt and jeans, which may have made them look slightly larger than they actually are. A frontal image for horizontal width and a right-side image for dorsoventral thickness were taken as the original images. These two original images were then distorted (−30, −10, +10 and +30%) in both width and thickness by using Adobe Photoshop CS4 (Adobe Systems, San Jose, CA, USA). The height of each image remained constant. The original image was presented 20 times, and each distorted image was presented 10 times, for a total of 120 images (60 images for each direction).

### Procedures

Participants were informed that their own and others' neck-down body images would be presented. Participants were asked to judge whether the presented image was of their own body or not by pressing two buttons on a four-button response pad with the right hand. They were also informed that all other participants wore an identical suit and that they were photographed in an identical posture to make the images indiscernible except for body size. Participants were instructed to judge the images based on body size. The buttons for self or others were counterbalanced between participants. E-prime software (Psychology Software Tools, Pittsburgh, PA, USA) was used to control the presentation of stimuli and to record responses.

Each image was presented for 1 s, with a mean interstimulus interval of 7 s (5.0, 7.0 or 9.0 s). The order of images was pseudo-randomized. After the experiment, participants were informed that all of the images they were shown were of their own original and distorted body, and they were asked whether they had noticed this.

### Imaging and data analysis

All brain images were acquired using a 3-T Siemens Trio magnetic resonance imaging (MRI) scanner (Munich, Germany). Functional images were obtained using a gradient-echo EPI sequence with the following parameters: 36 axial slices in the AC–PC plane; 2000 ms TR, 30 ms TE, 90° flip angle, 192 × 192 mm FOV, 3 × 3 mm in-plane resolution, 3-mm slice thickness, and no gap; the first six images were discarded. A T1-weighted anatomical scan of each participant was also obtained (1800 ms TR, 2.97 ms TE, 9° flip angle, 232 × 232 mm FOV, 1 × 1 mm in-plane resolution, and 1 mm slice thickness). Four-hundred and eighty-six scans were acquired for each participant.

Preprocessing and data analysis were performed using the spm8 software package (Wellcome Trust Centre for Neuroimaging, London, UK). Individual slices of a functional volume were temporally corrected for differences in acquisition time with reference to the middle (18th) slice. To correct for head motion, functional images of each participant were realigned with reference to the first image. Anatomical images were co-registered with the first functional images and normalized to the Montreal Neurological Institute (MNI) brain template. Functional data were then normalized using the same transformation parameters with a voxel size of 3 mm × 3 mm × 3 mm, smoothed in the spatial domain (isotropic Gaussian kernel of 8 mm full-width at half-maximum). Functional data were analysed using an event-related design. A general linear model was applied for each of the two orientations by five sizes and self–other judgments; the analysis was modeled using the canonical hemodynamic response function and its temporal derivative for each event type. Subjective ‘self’ and ‘others’ body perceptions associated with faster response times (RTs) were further analysed using parametric modulation. Random effects analysis was performed. Global scaling was not applied. Statistical parametric maps were generated for each contrast of the *t*-statistic on a voxel-by-voxel basis. These *t*-values were then transformed into *z*-scores in the standard normal distribution. The threshold of significance was set at *P* < 0.001, and more than 10 continuous voxels were reported. This threshold was uncorrected for multiple voxel-wise comparisons to balance the trade-off between Type I and Type II errors (Lieberman & Cunningham, 2009). MRIcron (http://www.mccauslandcenter.sc.edu/mricro/mricron/index.html) was used for image rendering.

To examine the brain regions involved in the perception of horizontal expansion, weightings of 0.7, 0.9, 1.0, 1.1 and 1.3 were applied to horizontal −30%, −10%, original, +10% and +30% images, respectively, and contrasted with weightings of 1.0 for each of the five dorsoventral images. For dorsoventral expansion, weightings of 0.7, 0.9, 1.0, 1.1 and 1.3 were used for −30%, −10%, original, +10% and +30% dorsoventral images, respectively, and contrasted with weightings of 1.0 for each of the five horizontal images. The two contrasts were calculated for each participant, and one-sample *t*-tests were conducted.

Neural responses to subjective own-body perception vs. that of other-body perception (combined horizontal and dorsoventral) based on individual responses were also examined using one-sample *t*-tests. Those subjective own- and other-body judgments associated with faster RTs were also analysed.

## Results

### Behavioral results

Mean responses for original and distorted images are shown in Table 1. Participants judged slightly reduced (−10%) images as their own body most frequently, both in width and thickness. Original size images were judged to be fatter, whereas skinny (−30%) images were judged to be moderately slimmer than themselves. Grossly oversized (+30%) images were mostly rejected as their own in both directions, whereas skinny width images were relatively accepted. Subjective own-body judgments were slightly higher for horizontal images than for dorsoventral images, although this difference was not statistically significant. Overestimations of own-body size were not observed.

**Table 1:** Mean responses (and SE) for original and distorted own body images

	−30%	−10%	Original	+10%	+30%
Horizontal width					
Own body perception	0.43 (0.13)	0.65 (0.07)	0.44 (0.08)	0.13 (0.06)	0.44 (0.02)
Mean RTs	1078 (228)	1300 (197)	1016 (111)	839 (123)	690 (130)
RTs for own perception	1590 (192)	980 (209)	964 (119)	1398 (115)	2051 (110)
RTs for other perception	807 (161)	1738 (180)	1027 (123)	701 (267)	518 (132)
Dorsoventral thickness					
Own body perception	0.18 (0.09)	0.60 (0.11)	0.36 (0.09)	0.13 (0.07)	0.00 (0.00)
Mean RTs	1209 (308)	1201 (208)	1155 (217)	766 (204)	446 (76)
RTs for own perception	2157 (476)	1143 (259)	1355 (191)	838 (216)	–
RTs for other perception	1122 (331)	1346 (306)	1072 (323)	744 (204)	446 (76)

RTs (ms).

A 2 × 5 anova [two orientations (width, thickness) × five body sizes (−30%, −10%, original, +10% and 30%)] for own-body judgment (arcsine values) revealed no differences between directions [*F*_1,10_ = 3.41, *P* = 0.09, not significant (NS)], but significant differences between sizes (*F*_4,40_ = 12.35, *P* < 0.001). *Post hoc* analyses (Ryan's method) showed that −10% distortions were judged as own-body more often than +30% (*t* = 6.38, *P* < 0.001), +10% (*t* = 4.99, *P* < 0.001) and −30% (*t* = 3.13, *P* < 0.005) distortions. Original images were judged as own-body more often than both +30% and +10% distortion images (*t* = 4.28, *P* < 0.001 and *t* = 2.89, *P* = 0.006, respectively).

A two-way anova for mean RTs showed no effect of image orientation (*F*_1,10_ = 0.008, *P* = 0.93, NS), suggesting no difference in task difficulty between width or thickness. However, the anova did reveal significant differences between sizes (*F*_4,40_ = 5.70, *P* = 0.001), and *post hoc* analyses revealed faster responses for +30% distortions than for −10%, −30% and original images (*t* = 4.22, *P* < 0.001; *t* = 3.56, *P* < 0.001; and *t* = 3.20, *P* = 0.003, respectively).

After the experiment, the participants were asked whether they had noticed that all images were of their own body, with no other bodies presented. Most participants (eight out of 11) were sure that other bodies had been presented. Three participants reported that they had suspected as much mid-way through the test; however, they were not convinced, and their performance was similar to that of the other participants.

### Functional MRI results

#### Activity associated with width

**FIG. 2:**
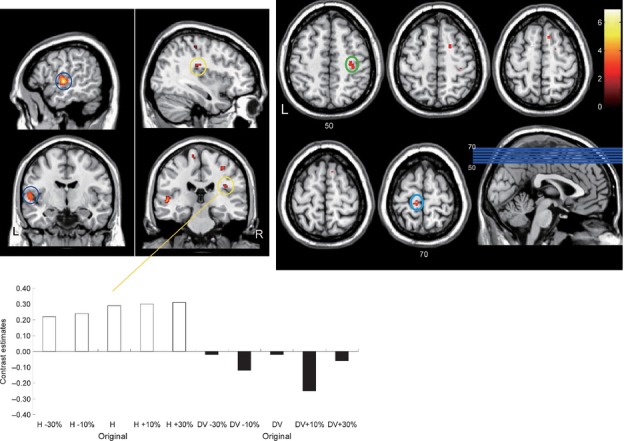
Brain regions involved in the perception of frontal body images and associated with expansion in the horizontal direction. L and R denote left and right, respectively. The left panel shows the bilateral parietal operculum (OP). Yellow circles indicate the right OP and blue circles indicate the left OP. In the bottom panel, the contrast estimates (beta parameters of the general liner model) for the right OP (MNI coordinates of *x*, *y*, *z*: 38, −22, 26; or a nearest peak) corresponding to each body image are shown. Primary somatosensory areas (SI) are shown in the right panel. Transverse slices of MNI coordinates of *z* 50–70 are shown. The green circle denotes the possible arm-to-shoulder region, and the light blue circle denotes the possible trunk-to-leg region. The color bar shows the *t*-value.

The brain regions that exhibited enhanced activity in relation to width and thickness images are shown in Table 2. In addition to the superior and middle temporal gyrus, the bilateral SI and secondary somatosensory cortex (SII) were also involved in the perception of body images and expansions in width. Left SII activity was located in the rostral (anterior) part of the lateral cortex, whereas right SII activity was more caudal (posterior) and medial (Fig. 2, left). The *z*-coordinate of activity in the right SI was 50, while the *z*-coordinate for activity in the left SI was 70 (Fig. 2, right). The right SI region was estimated to represent the arm-to-shoulder area, whereas the left SI was thought to possibly represent the trunk-to-leg area. The right anterior IPL was also involved in perceiving horizontal width.

**Table 2:** Regions showing enhanced activity corresponding to body sizes (−30%, −10%, original, +10%, +30%)

Region (BA)	MNI coordinates			*Z* value	Voxels
	
L/R	*x*	*y*	*z*		
Horizontal width					
L Secondary somatosensory cortex (43) & Superior temporal gyrus (22)	−55	−12	2	5.38*	234
R Superior temporal gyrus (22)	46	−32	11	5.25*	130
L Primary somatosensory cortex (3)	−12	−24	66	3.89	26
L Middle temporal gyrus (21)	−42	−44	8	3.89	10
R Secondary somatosensory cortex (43)	38	−20	25	3.88	16
R Posterior cingulate cortex (31)	−18	−58	12	3.86	36
R Pre-supplementary motor area (6)	10	20	56	3.73	24
R Inferior parietal lobule (39)	48	−54	16	3.72	14
R Primary somatosensory cortex (3)	32	−19	47	3.63	40
R Middle temporal gyrus (21)	51	−1	−13	3.49	15
R Supplementary motor area (6)	20	6	51	3.42	13
Dorsoventral thickness					
L Fusiform gyrus (36/37)	−32	−28	−22	3.89	16
R Visual cortex (18)	10	−70	7	3.84	40
R Inferior parietal lobule (40)	48	−39	42	3.29	10

BA, Brodmann area; L/R, Left/Right.

* Activation survived family wise error correction (*P* < 0.05) for whole brain multiple voxel-wise comparisons.

#### Activity associated with dorsoventral thickness

**FIG. 3:**
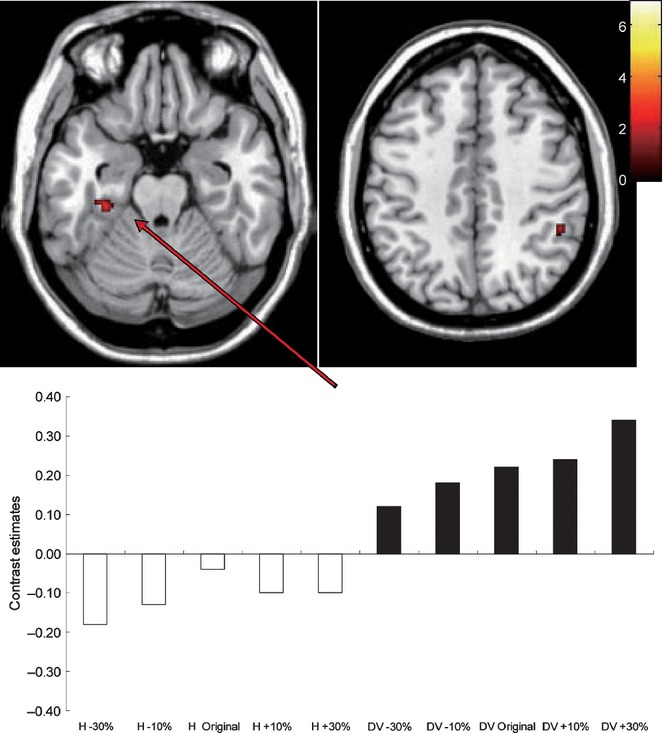
The left fusiform body area (FBA, left) and the right inferior parietal lobule (IPL; right) exhibited greater responses to body images of dorsoventral thickness than those of horizontal width. In the bottom panel, the contrast estimates for the FBA (MNI coordinates of *x*, *y*, *z*: −32, −28, −22; or a nearest peak) corresponding to each body image are shown. The color bar shows the *t*-value.

The left fusiform gyrus and the right IPL were associated with the perception of body images of dorsoventral thickness and their expansions (Fig. 3).

#### Own-body vs. other-body judgments

**FIG. 4:**
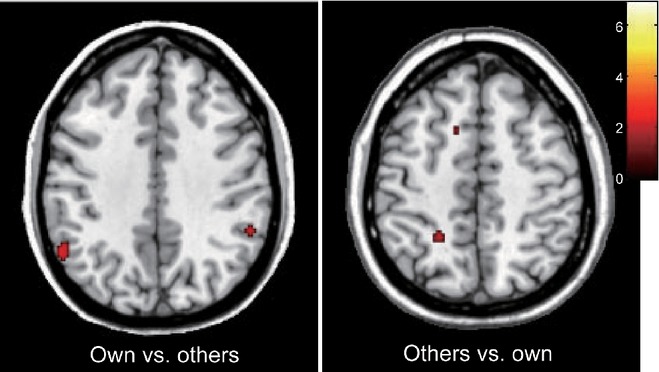
Regions associated with subjective perception of own and others' bodies. The bilateral inferior parietal lobule (IPL) showed enhanced activity for own body images (left). The left superior parietal lobule (SPL) and pre-supplementary motor area (SMA) were involved in judgment of others' body images (right). The color bar shows the *t*-value.

Subjective own-body perception of body images induced differential neural activity when compared with that induced by other-body perception (Table 3). Own-body perceptions were associated with the bilateral IPL (Fig. 4, left), whereas the left superior parietal lobule (SPL) and pre-supplementary motor area (pre-SMA) were involved in other-body perception (Fig. 4, right).

**Table 3:** Regions showing differential activity with regard to subjective perception of own and others' body

Region (BA)	MNI coordinates			*Z* value	Voxels
	
L/R	*x*	*y*	*z*		
Own > others					
L Inferior parietal lobule (40)	−55	−48	41	3.33	41
R Inferior parietal lobule (40)	53	−41	35	3.30	13
Others > own					
R Posterior cingulate cortex (23)	8	−53	25	4.09	38
R Superior parietal lobule (7)	−22	−44	48	3.77	13
L Supplementary motor area (6)	−14	8	47	3.32	10

BA, Brodmann area; L/R, Left/Right.

Faster RTs were associated with activation of the right insula (MNI coordinates: 34, −6, 16; *z-*value: 3.46; voxels: 16) for other than own-body perception. No effects of faster RTs on own-body perception were observed.

## Discussion

The neural mechanisms involved in the perception of body size were found to differ between the horizontal and dorsoventral directions. Perceiving wider bodies induced greater activity in somatosensory areas, whereas the perception of deeper bodies resulted in enhanced activity in the higher visual and posterior parietal areas. Bodily sensations could contribute more to frontal body appearance than visual processes. In addition, when compared with other-body judgment, own-body judgment yielded dissociated neural activity in the posterior parietal area. Therefore, body representation in the brain may be a perceptive and cognitive integration of distributed components.

Greater activity in the SI and SII was observed for body width rather than for dorsoventral thickness. Familiar frontal views of body images may induce bodily sensations. Moreover, somatosensory representations may contribute to slightly greater own-body judgments (Frassinetti *et al*., 2011) for horizontal images than for dorsoventral images. Possible representation of arm-to-shoulder and trunk-to-leg regions in the SI was detected in this study, but more participants are necessary to validate these results. Analogous to the SI, the SII has been shown to have a somatotopic body representation in humans (Disbrow *et al*., 2000). Tactile stimuli on the trunk induced activity in the anterior lateral regions of the SII, whereas stimuli on the legs yield posterior medial activity (Eickhoff *et al*., 2007). These regions overlap with those observed in our study for width perception and are consistent with estimated body parts in the SI. The anterior lateral region of the SII is closely integrated with basic sensorimotor areas (Eickhoff *et al*., 2010). In contrast, the right posterior medial portion of the SII activated in this study may be the PIVC (Eickhoff *et al*., 2006) that has been reported to be involved in own-body perception (Lopez *et al*., 2008). Somatosensory sensation of the lower limbs may be associated with vestibular sense (Horak & Hlavacka, 2001) and may also be associated with body-width perception.

The area of the fusiform gyrus found to be associated with perceiving dorsoventral body thickness in this study may be the body-selective area, namely, the FBA (Peelen & Downing, 2005; Schwarzlose *et al*., 2005). Although the percentage changes in each dimension are the same, the absolute change in the dorsoventral dimension will be smaller than the absolute change in width. Therefore, distortions in thickness may require finer visual processing for dorsoventral images than horizontal images. Because the left anterior fusiform gyrus is involved in visuo-haptic processing (Kim & James, 2010), participants may touch their leg or trunk subconsciously to compare the thickness of their own body with that shown in the presented images. In addition, neuroimaging studies (Astafiev *et al*., 2004; Peelen & Downing, 2007) suggest that body-part images are processed in the extrastriate body area (EBA), which may be used to differentiate own-body images from other-body images (Vocks *et al*., 2010). Consistent with our results, no differences in EBA activity were observed between own-body and other-body judgments (Hodzic *et al*., 2009). The FBA may be involved in whole-body perception (Taylor *et al*., 2007), whereas the EBA may be associated with body-part processing. The left anterior fusiform gyrus activation observed in this study was more anterior than the right FBA activity reported in a previous study (Moro *et al*., 2008). Therefore, future studies reporting on the activation of the left FBA are required to discuss appropriate coordinates.

The dorsoventral image is orthogonal to the horizontal image and *vice versa*. The differences between horizontal and dorsoventral images found in this study may be derived from viewpoint differences. Body images rotated by 0–45° induced repetitive neuronal reduction in the right FBA, whereas those rotated by 60° were processed differently (Taylor *et al*., 2010). Therefore, the enhanced FBA activity observed for dorsoventral thickness, rather than width, in this study may suggest the presence of separate processing modes. Moreover, instead of physically changing the viewpoint, mental rotation can be applied to either view. This mental rotation process may be related to the judgment of others' bodies. The enhanced superior parietal activity observed in this study for other-body judgments may be associated with mental rotation (Gogos *et al*., 2010). Allocentric visual body representation may be associated with the left SPL (Corradi-Dell'Acqua *et al*., 2009).

Consistent with other lesion and neuroimaging studies, we observed posterior parietal cortex involvement in body perception. The right parietal cortex is associated with the conscious perception of the body, whereas left parietal activity is involved in monitoring action (Daprati *et al*., 2010). Multisensory integration in the inferior parietal area can represent body ownership (Tsakiris, 2010). Probabilistic fiber tract analysis in human inferior parietal areas shows that rostral/anterior portions (supramarginal gyrus) have strong connectivity with somatosensory areas, whereas caudal/posterior portions (angular gyrus) have connectivity with temporal areas (Caspers *et al*., 2011). Our finding of bilateral inferior parietal activity during subjective own-body perception may reflect sensorimotor representation and multisensory integration.

In patients with anorexia nervosa, alterations in IPL activity have been reported (Pietrini *et al*., 2011; Gaudio & Quattrocchi, 2012). These patients also display reduced regional brain volumes in the right anterior insular cortex, bilateral parahippocampal gyrus and left fusiform gyrus (Brooks *et al*., 2011). It has also been demonstrated that visuospatial and somatosensory functional connectivity in patients with anorexia nervosa is disrupted (Favaro *et al*., 2012). Thus, it is possible that the impaired neural networks observed in anorexic patients could also be involved in the differential perception of body size observed in this study.

The sense of body ownership has been studied using illusory tactile attribution to visual body-like objects, and sensorimotor inputs can facilitate visual attribution of body ownership associated with the premotor cortex (Petkova *et al*., 2011; Zeller *et al*., 2011). Meanwhile, as shown in this study, a lack of sensorimotor inputs and an indiscernible view may require the utilization of proprioceptive information for implicit own-body perception (Frassinetti *et al*., 2011). In contrast, denying own-body perception (i.e. other-body judgments in this study) may inhibit such somatosensory information. The pre-SMA is involved in unconscious inhibition (van Gaal *et al*., 2010), and is also associated with self/other differentiation in action (Ferri *et al*., 2012). The pre-SMA activity involved in other-body judgments in this study may inhibit implicit body movement to differentiate self from other. In addition, the right insula activation associated with faster responses for other-body might be involved in negative feelings (Harrison *et al*., 2010) for bodies unacceptable as own.

We observed that slightly slimmer body images were most acceptable as own-body images in this study. On the contrary, distorted images of the hand appear to be less accepted if they depict a shrunken view than if they depict an enlarged view (Pavani & Zampini, 2007; Marino *et al*., 2010). Hands (and face parts), which occupy a large cortical area, may induce differential sensations from other body parts when they are visually distorted. An obscure whole-body image, in contrast to a localized and detailed hand image, may be underestimated. In addition, subjective and explicit self/other-body perception (Sforza *et al*., 2010; Mazzurega *et al*., 2011) may be dissociated from implicit self–other judgments (Frassinetti *et al*., 2008; Richetin *et al*., 2012). Our result of self–other dissociation may be dependent on the task and stimuli. Harsh judgments for body images in anorexic patients (Smeets, 1999) may reflect alterations in acceptability for body sizes. Other-body processing in this study may have more to do with the acceptance of a given body distortion than real self–other distinction.

Visual size can vary depending on distance and angle. Therefore, the size of a person's own body in the mirror is a relative feature. Our finding of moderate underestimation of own-body size may be derived from these factors. Although binocular disparity is essential for depth perception, the close proximity of one's own body under direct vision may prevent the utilization of visual depth information. However, somatosensory information is more robust, even though it is not as precise as vision. Blocking somatosensory information with anesthesia induces the sensation of swelling both in large and small body parts (Gandevia & Phegan, 1999; Paqueron *et al*., 2003). These results suggest that somatosensory information is necessary for defining body size and shape even if vision is available. A person may need proprioceptive sensation and position information of body parts to recognize their own body.

Although gender differences in brain anatomy (Luders & Toga, 2010) and hormones (McCarthy *et al*., 2009) may limit the generalization of our results, the use of women in this type of study is preferable because they are highly body-size conscious (Wardle *et al*., 2006). The majority of patients with anorexia nervosa are women and, when compared with healthy women, these patients show reduced brain activity in response to images of their own and other bodies (Sachdev *et al*., 2008). Additionally, significant gender differences in weight assessment (Christensen, 2012) may affect body-size perception. Size overestimation of bodies and objects in the left hemisphere of both men and women, as well as a right hemispheric contribution to body-size overestimation in women, have been reported (Mohr *et al*., 2007). These results suggest that body-size perception in men and women needs to be examined separately. Further studies examining gender effects on body-size perception in both horizontal and dorsoventral directions may be fruitful.

In conclusion, the neural substrates involved in the processing of frontal and side views of identical body images differ in the human cortex. Visual and somatosensory processes and their integration may construct a whole-body representation and may be responsible for the dissociation of own-body perception from other-body perception.

## Abbreviations

EBA: extrastriate body area
FBA: fusiform body area
IPL: inferior parietal lobule
MNI: Montreal Neurological Institute
PIVC: parieto-vestibular cortex
RTs: response times
SI: primary somatosensory cortex
SII: secondary somatosensory cortex
SMA: supplementary motor area
SPL: superior parietal lobule
